# Innate Lymphoid Cells: Important Regulators of Host–Bacteria Interaction for Border Defense

**DOI:** 10.3390/microorganisms8091342

**Published:** 2020-09-02

**Authors:** Katharina Beck, Hiroshi Ohno, Naoko Satoh-Takayama

**Affiliations:** 1 Laboratory for Intestinal Ecosystem, Center for Integrative Medical Sciences, RIKEN, 1-7-22 Suehiro-cho, Tsurumi-ku, Yokohama 230-0045, Japan; katharina.beck@riken.jp (K.B.); hiroshi.ohno@riken.jp (H.O.); 2 Immunobiology Laboratory, Graduate School of Medical Life Science, Yokohama City University, 1-7-29 Suehiro-cho, Tsurumi-ku, Yokohama 230-0045, Japan; 3 Intestinal Microbiota Project, Kanagawa Institute of Industrial Science and Technology, 3-2-1 Sakado, Takatsu-ku, Kawasaki 213-0012, Japan; 4 Laboratory for Immune Regulation, Graduate School of Medicine, Chiba University, 1-8-1 Inohana, Chuo-ku, Chiba 260-8670, Japan

**Keywords:** innate lymphoid cells (ILCs), bacterial infection, mucosal defense, microbiota, intestinal homeostasis

## Abstract

Innate lymphoid cells (ILCs) are a recently discovered type of innate immune lymphocyte. They include three different groups classified by the nature of the transcription factors required for their development and by the cytokines they produce. ILCs mainly reside in tissues close to the mucosal barrier such as the respiratory and gastrointestinal tracts. Due to their close proximity to the mucosal surface, ILCs are exposed to a variety of both commensal and pathogenic bacteria. Under non-pathological conditions, ILCs have been shown to be important regulators for the maintenance of tissue homeostasis by mutual interactions with the microbiome. Besides these important functions at homeostasis, several studies have also provided emerging evidence that ILCs contribute to defense against pathogenic bacterial infection by responding rapidly to the pathogens as well as orchestrating other immune cells. In this review, we summarize recent advances in our understanding of the interactions of ILCs and bacteria, with special focus on the function of the different ILC subsets in bacterial infections.

## 1. Introduction

Mucosal surfaces are confronted with an enormous number of both commensal and pathogenic bacteria, and the immune system has evolved various mechanisms for maintaining and shaping the indispensable symbiosis between the host and the microbiota. However, some bacteria have devised mechanisms to overcome the host protective responses and thereby can cause severe infections. In recent years, innate lymphoid cells (ILCs) have been shown to play a critical role in maintaining homeostasis and in protective immunity, as well as being important mediators between the microbiota and the adaptive immune system. ILCs are integrated in all sites of the mucosal tissues, although a limited group of ILCs can be found in almost all organs. ILC-mediated defense is characterized by a rapid reaction achieved by cytokine release. Here we will describe how the responses of ILCs are regulated by microorganisms, including non-pathogenic bacteria, as well as their functional role in bacteria-triggered diseases.

## 2. Definition of ILC Subsets and General Functions

All ILCs originate from a common innate lymphoid progenitor (CLP) and the later stage ILC progenitor cell (ILCP). Based on their differentiation and function, ILCs can be divided into three subgroups: ILC1, ILC2, and ILC3 which are considered as innate analogues of the three major CD4^+^ T effector cells, Th1, Th2, and Th17 because of their similar functions [1]. Unlike these adaptive T cells, ILCs do not express antigen receptors, but instead they display several activating and inhibitory receptors. ILCs can be subdivided based on the key transcription factors that control their cytokine profiles and signature functions.

By analogy to Th1 cells, ILC1 mediate type 1 immunity against tumors and intracellular pathogens such as viruses and certain bacteria. ILC1 mediate their effects through interferon gamma (IFN-γ) and tumor necrosis factor (TNF)-α cytokine production, which depends on the transcription factor T-box expressed in T cells (T-bet) [1]. Initially, it has been suggested to include conventional natural killer (cNK) cells in the same group as ILC1 [2], but now they are defined as two groups [1]. NK cells differentiate from a common innate progenitor (CILP) but differ from ILC1 in their dependence on eomesodermin (EOMES) during differentiation [1,3,4]. Similar to ILC1, cNK express IFN-γ but additionally they produce perforin and granzyme. Both ILC1 and cNK cells function collaboratively as the innate counterparts of cytotoxic CD8^+^ T cells [5].

ILC2 mediate a type 2 immune response, which is characterized by their production of the cytokines IL-4, IL-5, IL-9, and IL-13, as well as amphiregulin [6,7,8]. The function and development of ILC2 rely on the expression of the transcription factor GATA3 and RORα [9,10,11]. They express the IL-33 receptor component ST2, which provides the basis for their activation by IL-25 and IL-33. ILC2 have recently been shown to play a crucial role in defense against extracellular parasites [12,13,14], in allergic-responses [15] and in tissue repair [16,17]. Dysregulation of ILC2 is associated with allergic diseases like asthma and atopic dermatitis [18].

ILC3 are characterized as the Th17 equivalent innate immune cells due to their production of IL-22 and/or IL-17. ILC3 are highly abundant in the intestine where they play a crucial role in intestinal immunity including the maintenance of mucosal barrier integrity and microbiota-host homeostasis. It is well-documented that retinoic acid-related orphan receptor gamma t (RORγt) is essential for ILC3 development and is indispensable for ILC3 determination [1,19,20]. ILC3 can be divided into at least three subpopulations based on the expression of NKp46, also known as natural cytotoxic receptor 1 (NCR1), and CCR6. NKp46^+^CCR6^−^ILC3 mainly produce IL-22 rather than IL-17 in response to IL-23 and IL-1β [21]. In contrast, NKp46^−^CCR6^+^ ILC3, known as lymphoid tissue inducer (LTi) cells due to their functional contribution to lymphoid organ formation, produce both IL-22 and IL-17 and additionally lymphotoxins [21]. LTi cells derive from an earlier common progenitor (CHILP) but differ in the developmental path from other ILC3 [1,3,4]. The third subgroup lacking CCR6 and NKp46 expression is a mixed population, which additionally co-express the transcription factor T-bet and also includes ex-NKp46^+^ ILC3 [22,23].

Even though ILCs are characterized by the expression of specific transcription factors, lineage tracing studies have provided clear evidence that there is plasticity among the ILC subgroups [1,24]. It has been shown that ILCs have an ability to adapt to the local environment by changing their transcription profiles. ILCs, including NK cells, are initially derived from the same progenitor. Circulating naïve ILCs, which are referred to as CD127^+^CD117^+^ ILCs, were recently found in human peripheral blood but can also be found in the tissue where they may go through further maturation [25]. The capacity to re-shape the subtypes upon microenvironmental changes and specific cytokine exposure has been shown to result in changes in ILC identity and in functional capacity. For instance, NKp46^−^CCR6^−^ double negative ILC3 are able to convert into NK1.1^+^NKp46^+^ ILC1 [22,26,27]. Moreover, the conversion of ILC2 into IFN-γ-producing ILC1 has been reported recently [28,29,30]. This capacity for plasticity might be the reason for the complex heterogeneity of ILCs, complicating the identification of the specific role of each subgroup in diseases.

Murine ILC subsets have been widely shown to be tissue-specific due to the organ-specific microenvironment [31,32]. The spatial distribution of ILCs in humans and mice is dissimilar and big efforts have been made to specify the ILC distribution in human non-pathological conditions [33,34]. Especially ILC1 showed brought heterogeneity throughout the tissues [33], thus the distribution of ILCs in different organs/tissues requires further studies. Here, we mainly review the function of mature ILCs, which are mostly tissue-resident in each organ, with a special focus on bacterial infection or colonization.

## 3. Infections of the Gastrointestinal Tract

### 3.1. ILC1 and NK Cells

ILC1, including NK cells, are mainly involved in the defense against tumors and intracellular pathogens such as viruses. Information about the interaction of ILC1 with bacteria had so far been limited, but has become better understood because of several recent reports [1,35,36,37,38,39,40,41,42,43,44,45,46,47]. A human study in which colonic lamina propria-derived ILC1 were exposed to commensal gut bacteria, interestingly showed that only Gram-negative bacteria induced the production of IFN-γ. CD11c^+^ myeloid dendritic cells (mDC) mediate ILC1 stimulation by releasing the cytokines IL-12p70, IL-18, and IL-1β, resulting in ILC1-derived production of granzyme B and IFN-γ [35]. Moreover, in vitro experiments indicated that IFN-γ could promote the translocation of *Escherichia coli (E.coli)* due to cytokine-dependent tight junction disruption [36]. Consequently, ILC1 are currently thought to have a high priority in defense against pathogenic bacteria and are mainly associated with bacterial infections of the gastrointestinal tract.

#### 3.1.1. *Citrobacter rodentium*

Infection with the extracellular enteric mouse-specific pathogen *Citrobacter rodentium* (*C. rodentium*) leads to gastrointestinal lesions in the gut mucosa that are similar to symptoms caused by the human pathogens enteropathogenic *E. coli* (EPEC) and enterohaemorrhagic *E. coli* (EHEC). Consequently, *C. rodentium* has been widely used as a model to study the host response to infections as well as the interactions of pathogen, host and microbiota [37]. *C. rodentium* infection in mice affects the caecum and the colon, where it causes acute inflammation and crypt hyperplasia.

IFN-γ producing ILC1 and NK cells have been shown to be important for protection against *C. rodentium* infection. Indeed, NK cell-depleted mice had a higher bacterial load accompanied by more severe inflammation. They also had lower levels of IFN-γ, TNF-α, IL-12, and pathogen-specific IgG in the colon, and a delay in homing of IFN-γ^+^ CD4^+^ T cells to the intestine. These findings indicate that NK cells promote bacterial clearance through antibody production against *C. rodentium* [38]. Moreover, *C. rodentium* infection leads to expanded ILC1 number in the small intestine [39], which might be due to ILC1/3 plasticity in the gut.

#### 3.1.2. *Clostridium difficile*

Infections with *Clostridium difficile* (*C. difficile*) mainly occur in patients with disturbed gut microbiota as a consequence of broad-spectrum antibiotics therapy [40]. Pathogenesis evolves by ingestion of *C. difficile* spores, which germinate in the presence of bile acids and other signals like Ca^2+^ and amino acid derivates in the dysbiotic gut. The bacteria colonize and adhere to the epithelial cells, where they produce toxin A and B and, in some strains, binary toxin ‘CDT’. This leads to the onset of disease due to disruption of the actin cytoskeleton, epithelial cell rounding and cell death. The infection causes a wide spectrum of symptoms ranging from mild diarrhea to toxic megacolon and even death [40,41].

A study using *C. difficile-*infected C57BL/6*Rag1*^−/−^ mice, which lack T and B cells, showed upregulation of ILC1 and ILC3 associated proteins such as IFN-γ, TNF-α and Nos2 (ILC1-derived) as well as IL-22, IL-17a, and RegIIIγ (ILC3-derived). The further analysis of *Rag2*^−/−^*Il2rg*^−/−^ (also termed *Ra^−/−^γc^−/−^*) mice, which also lack all innate lymphoid cells, showed increased infection susceptibility to *C. difficile*, which could be rescued by ILC transfer. Especially, the loss of IFN-γ or T-bet-expressing ILC1 in *Rag1*^−/−^ mice was associated with increased infection susceptibility, revealing a protective role of ILC1 in *C. difficile* infection [42].

#### 3.1.3. *Salmonella* spp.

*Salmonella typhimurium* (*S. typhimurium*) and *S. enteritidis* are pathogenic bacteria that cause gastroenteritis, mostly after the ingestion of contaminated food. The symptoms include nausea, vomiting, fever, diarrhea, and cramping. The infection usually self-resolves after several days, which might be one reason for the lack of human studies on the role of ILCs in *S. typhimurium* infection. In the mouse model, *S. typhimurium* infection can only be induced by prior streptomycin treatment [43]. Immune defense against *S. typhimurium* infection is characterized by increased IFN-γ secretion [44], which induces the defense mechanisms of other immune cells [45] and also helps to stimulate mucus secretion from goblet cells [46]. Loss of NK cells and the subsequent absence of protective IFN-γ secretion has been shown to increase susceptibility in the murine *S. typhimurium* infection model [44,47]. The study by Castleman et al. [35] further indicated that the in vitro exposure of commensal Gram-negative and pathogenic *S. typhimurium* to human colonic lamina propria cells led to increased IFN-γ expression by ILC1 and NK cells, which was mediated by the cytokines IL-12p70, IL-18, and IL-1β [22].

### 3.2. ILC2

In the past, ILC2 have been mainly associated with wound repair and helminth infections rather than bacterial infections [48,49,50]. Through induction of goblet cell differentiation, they promote mucin secretion and, thereby, strengthening the secondary barrier of the intestine against pathogens [51,52]. However, more recently ILC2 are also gaining attention for their role during bacterial infection. ILC2-activating factors such as TSLP are influenced by the microbiota, thus suggesting possible interaction with and modulation of ILC2 functions [53]. An in vitro study on human circulating ILC2 showed that ILC2 express Toll-like receptors (TLR) 1, 4, and 6. Upon TLR stimulation, ILC2 induced immunoglobulin (IgM, IgG, IgA, and IgE) production by co-cultured B cells, which in turn shaped the composition of the microbiota [54]. We recently reported a similar mechanism for ILC2 in the stomach, where ILC2 are the most abundant ILC [55].

#### 3.2.1. *Clostridium difficile*

As mentioned above, *C. difficile* infection leads to epithelial damage due to the release of toxins and mainly involves the ILC1 and ILC3 subsets. However, recent studies further reveal the importance of ILC2 for *C. difficile* infection prognosis. Frisbee et al. observed upregulation of IL-33 production during *C. difficile* infection, both in mice and humans [56]. IL-33 activates ILC2, which further protect from toxin-mediated epithelial damage via recruitment of eosinophils. *Rag^−/−^γc^−/−^* mice showed a worse prognosis after *C. difficile* infection, which could be reversed by ex vivo transfer of ILC2. These reports suggested that ILC2 activation, triggered by IL-33, is also involved as an important defense mechanism against *C. difficile*-derived colitis [56].

#### 3.2.2. *Helicobacter pylori*

Gastric *Helicobacter pylori (H. pylori)* infection is highly prevalent in humans. Chronic infection has been associated with the development of pathologies such as gastric ulcers and gastric cancer. It has been recently observed that *H. pylori* infection results in an increased ILC2 population in human and mouse stomach tissue, accompanied by elevated IL-5 production and B cell numbers [55,57]. Treatment of *H. pylori*-infected germ free (GF) mice with an anti-IL-5 neutralizing antibody significantly decreased the B cell population. ILC2-mediated B cell activation enhanced the IgA response against *H. pylori* and subsequently provided protection against the infection. Even though only a minor role in homeostasis regulation can be attributed to ILC2, they have recently been shown to be involved in the defense against several pathogens. ILC2-deficient mice suffered from exacerbated inflammation and gastric bleeding [55]. At present, increasing antibiotic resistance among *H. pylori* strains is causing problems in patients [58]. The new insights into *H. pylori* infection and the underlying mechanism of immunity may lead to better approaches for therapy.

### 3.3. ILC3

ILC3 has been understood as the most important cell type involved in the repulsion of bacterial infection as well as in the interaction with commensal microbiota. The interaction with commensal bacteria has been addressed by many groups; however, the exact role of ILC3 remains controversial. Whereas several studies showed a decreased number of ILC3 in GF mice [59,60], others did not observe a microbiota-mediated influence on the ILC3 population [61]. ILC3 sense various environmental signals including neuronal signals [62], as well as dietary and bacterial metabolites [63,64], which modulate ILC3 differentiation and function. Commensal bacteria periodically penetrate the mucosal barrier; thus, mechanisms have evolved to minimize inflammation and maintain physiological homeostasis. ILC3 has been reported to interact with commensal bacteria indirectly [65,66]. Interposed mononuclear phagocytes, such as CD11c^+^ DC, respond to commensal bacteria via secretion of IL-23 and IL-1β, which then induce IL-22 production by ILC3 [67]. ILC3-derived IL-22 is crucial for containing commensal bacterial dissemination through the induction of antimicrobial peptides such as RegIIIβ, RegIIIγ, and peptides of the S100 family [68]. Depletion of ILCs leads to systemic inflammation [69]. IL-22 signaling further acts on intestinal epithelial cells by inducing fucosylation [70], thus maintaining the barrier integrity in the intestinal tract. It has been also reported that CCR6-expressing lymphoid tissue inducer (LTi)-like ILC3 express major histocompatibility complex class II (MHC class II) to downregulate commensal bacteria-specific CD4^+^ T cell responses, and thus to limit spontaneous inflammation [71].

#### 3.3.1. *Citrobacter rodentium*

In the initial phase of *C. rodentium* infection, IL-22 expression by ILC3 is crucial for the immune defense [60,72], whereas clearance of the bacteria requires lymphocytes from the adaptive immune system [73]. Therefore, CD4^+^ cells are the main source of IL-22 in the late phase of infection [74,75]. Activation of ILC3 during *C. rodentium* infection is mediated by many pathways including specific activation of ILC3 surface receptors [59,76,77,78,79,80,81,82,83,84], diet- and bacteria-derived metabolites [85,86,87,88,89,90,91] and interposed phagocytes [92,93,94,95,96].

A recent study showed that knockout mice specifically lacking MHC class II on ILC3 had an increased Tfh cell response upon *C. rodentium* infection. This was accompanied by increased IgA levels as well as increased pathogen-specific IgA. Interestingly, the MHC class II depletion had no significant effect on the overall course of infection, indicating a crucial role of LTi-ILC3 in the orchestration of the IgA response [76]. In addition, ILC3 also express the NK cell receptor P1 (NKR-P1R), which is activated by C-type lectin-related type II transmembrane (Clr) proteins in the gut lamina propria. Loss of NKR-P1R leads to elevated numbers of ILC3, but increases susceptibility to *C. rodentium* due to impaired cell function [77].

The increased susceptibility to *C. rodentium* infection is also induced by disturbance of the gut microbiota, and thus dietary-derived metabolites presumably shape the immune system and further alter infection prognosis [97]. G-protein-coupled receptors (GPCR) are known as an important class of receptors for processing environmental cues. They are expressed on various immune cells and play an important role in cell recruitment and migration [97,98,99]. In recent years, the GPCR on ILC3 have been hypothesized to be involved in ILC3 regulation. Indeed, the GPC receptor GPR183 (also known as EBI2) is expressed on LTi-ILC3 cells. GPR183 activation by 7a,25-dihydroxycholesterol (7a,25-OHC), which is produced by stromal cells in lymphoid follicles, promotes ILC3 migration and is crucial for the formation of isolated lymphoid follicles and cryptopatches [78]. These findings were extended by another study, which demonstrated that ILC3 in the mesenteric lymph node (mLN) and lamina propria migrate towards the GPR183 ligand 7a,25-OHC in vitro. GPR183-mediated accumulation of ILC3 was thus crucial for optimal IL-22 production and protection against *C. rodentium* [79]. Another group additionally demonstrated the importance of the GPCR free fatty acid receptor 2 (Ffar2) in protection from bacterial infection [59]. Mice lacking Ffar2 showed exacerbated symptoms upon *C. rodentium* infection due to a decreased number of IL-22-producing ILC3 and an altered expression level of anti-bacterial peptides and mucus-associated proteins [59]. These reports indicate that various receptors, which are expressed on ILC3 and induced by the trigger of *C. rodentium* exposure, are involved in the regulation of immune responses.

The aryl hydrocarbon receptor (Ahr) is a ligand-inducible transcription factor that is expressed on various cell types in the gastrointestinal tract. There, it responds to environmental toxins such as 2,3,7,8-tetrachloro-dibenzo-p-dioxin (TCDD) but also to endogenous ligands from the diet, microbiome or host cells [80,100]. However, it has been reported that Ahr is expressed on ILC3, where it plays an important role in protection from *C. rodentium* infection, as Ahr-deficient mice showed augmented infection symptoms [81,82,83]. Ahr is also involved in the regulation of Th17 cells via ILC3 [71,84], suggesting a link between nutrients and ILC3, mirroring adaptive Th17 cells in the gut.

Another dietary-sensing molecule is vitamin A, which is widely associated with intestinal homeostasis by promoting mucosal barrier integrity and immune cell recruitment [101,102]. Moreover, the vitamin A metabolite retinoic acid (RA) is involved in the regulation of ILC3 activity. RA directly regulates the transcription factor RORγt and further ensures the correct development of ILC3 [85]. Lack of RA resulted in a diminished population of ILC3 and enhanced susceptibility to *C. rodentium* infection [86,87]. Recently, it has been also shown that ILCs express the transcriptional repressor, hypermethylated in cancer 1 (HIC1, ZBTB29), which is regulated by vitamin A. Lack of HIC1 abolished IL-22-expressing ILC3 and increased susceptibility to *C. rodentium* infection [88]. In contrast to vitamin A, vitamin D has been shown to negatively regulate ILC3 in *C. rodentium* infection. Mice lacking the vitamin D receptor (VDR) had increased resistance to *C. rodentium* infection, accompanied by an augmented number of ILC3 as well as increased IL-22 and anti-bacterial peptide secretion [89]. Subsequent responses to vitamin D suppressed IL-22, IL-17A, and GM-CSF secretion [90]. Consistent with these mouse studies, IL-23 and IL-1β stimulation of human mucosal ILC3 resulted in upregulation of the VDR. However, in contrast to these studies, a protective role of vitamin D in *C. rodentium* infection has been recently reported [91]. Here, knockout mice for the vitamin D 1α-hydroxylase (Cyp27B1), which converts vitamin D to the active VDR-binding form, developed a fatal *C. rodentium* infections course, characterized by reduced *Citrobacter*-specific antibody responses, diminished ILC3 and concomitant IL-22 and IL-17 expression [91].

Besides diet metabolites, a myeloid-ILC3 crosstalk has been proposed to influence the immune response in bacterial infection. Depletion of the chemokine receptor CX3CR1 lead to decreased IL-22 production by ILC3 and disturbed *C. rodentium* defense [92]. Upon infection, CX3CR1^+^ DCs release the chemokine CXCL16, which activates ILC3 via CXCR6, thus stimulating IL-22 release and the expression of antimicrobial peptides (AMPs) [93,94]. Expression of the tumor necrosis factor (TNF) family member TL1A in macrophages has been further shown to enhance IL-22 production by ILC3 [94]. Recently, the recombination signal binding protein for immunoglobulin Jκ (RBP-J), originally called that because it bound to the recombination signal sequences (RSS) upstream of each Jκ gene segment [103], has also been identified to be crucial for *C. rodentium* clearance by promoting the secretion of IL-22 by ILC3 via macrophages [95]. Moreover, studies on mice lacking β2-integrins using CD18^−/−^ mice revealed that β2-integrin expression on macrophages promotes ILC3-derived IL-22 expression via IL-1β, and thus is important for *C. rodentium* infection [96].

#### 3.3.2. *Clostridium difficile*

As mentioned above, *C. difficile* infection in *Rag1*^−/−^ mice revealed upregulation of both ILC1- and ILC3-associated proteins [42]. Loss of ILC3 or ILC3-derived IL-22 modestly reduced the resistance to acute *C. difficile* infection. A more recent study reported that the immune response of ILC3 during *C. difficile* infection can be enhanced by acetate, which is one of the short-chain fatty acids (SCFA) recognized by the cognate receptor, free fatty acid receptor 2 (Ffar2) [104]. Beyond the acetate-Ffar2 interaction, the enhanced expression of the IL-1 receptor on ILC3 boosts IL-22 secretion in response to IL-1β, which is increased in neutrophils during *C. difficile* infection.

#### 3.3.3. *Salmonella* spp.

*Salmonella* spp. are also major bacteria used for the functional analysis of ILC3 in mice. Many groups have found that ILC3-derived IL-22 is essential for protection against *S. typhimurium* infection [45,105]. ILC3 also induce epithelial fucosylation via *Fut2* expression in an IL-22- and lymphotoxin-dependent manner. Fucosylated carbohydrate moieties on intestinal epithelial cells form part of the environmental niche for commensal bacteria [70,106,107]. Thus, increased fucosylation strengthens the defense against *S. typhimurium* and concurrent shaping of the microenvironment [70].

As described above, the vitamin A-derived metabolite retinoic acid (RA) from intestinal epithelial cells promotes innate immunity by maturation and proliferation of IL-22-producing RORγt^+^ ILC3 [105,108]. The retinol dehydrogenase 7 (*Rdh7*) is the key enzyme for the vitamin A metabolism which catalyzes the conversion of retinol into RA [108]. *Rdh7* is known to regulate IL-22 expression by direct binding to the *il22* locus [63]. *Rdh7*-depleted mice showed a decreased number of NKp46^+^ ILC3 in the small intestine and diminished IL-22 expression, which in turn impeded the IL-22-dependent production of antimicrobial peptides such as RegIIIγ, RegIIIβ, and calprotectin subunits, S100A8 and S100A9. *S. typhimurium* infection in *Rdh7* knockout mice leads to significantly reduced pathogen load in the feces compared to control mice, revealing that RA promotes *S. typhimurium* colonization by shaping the microbial environment [109].

#### 3.3.4. *Helicobacter* spp.

The non-gastric species *Helicobacter apodemus* (*H. apodemus*) and *H. typhlonius* mainly colonize the intestine and have been shown to induce T cell responses [110,111]. A recent study using immune-compromised *Rag1^−/−^* mice showed that *Helicobacter* spp. negatively regulated RORγt^+^ ILC3, especially T-bet^+^ ILC3, and reduced their proliferative capacity. In contrast, wild type mice could sustain ILC3 function in the presence of *Helicobacter* spp., indicating an interaction between ILCs and effector T cells [112]. Hepworth and colleagues recently reported that ILC3 suppress the T cell-dependent IgA response to *H. typhlonius* in order to preserve the microbial niche [76]. Both studies revealed a mutual interaction of *Helicobacter* spp. and ILCs through a complex mechanism, which also involves adaptive T cell activation.

## 4. Infections of the Lung

The lung is a nonlymphoid organ, but also an organ at risk for infection due to its large exposure to antigens and pathogens, similar to the gastrointestinal tract. The lung exhibits an interconnected network of resident immune cells, thus many reports with a focus on lung immunity can be found currently. Initially, the important function of NK cells in the lung was discovered in both mouse and human [113,114]. In recent years, IL-33 produced by lung epithelium has been identified to be an important mediator of lung homeostasis. Immediate upregulation of IL-33 resulted in the recruitment of ILC2 and simultaneous appearance of alveolar macrophages [115]. It is generally understood that ILCs in the lung are mainly tissue-resident cells, which are strongly affected by changes in the local microenvironment.

### 4.1. ILC1 and NK Cells

#### 4.1.1. Pneumoniae

Pneumonia remains one of the most common causes of death upon infection in developed countries [116]. Lung infections are frequently caused by the pathogens *Streptococcus pneumoniae (S. pneumoniae), Klebsiella pneumoniae* (*K. pneumoniae*), and *Pseudomonas aeruginosa* (*P. aeruginosa)*. Innate lymphocyte-derived inflammatory responses including TNF, IL-23, IL-17, and IFN-γ has been shown to be essential for the clearance of *K. pneumoniae* infection [117,118]. A mutual interaction between NK cells and macrophages play a critical role for bacteria containment. Macrophage-derived type I IFNs promote the production of IFN-γ in NK cells, which in turn induces a feed-back loop to produce IL-12 in macrophages [119].

#### 4.1.2. *Bordetella pertussis*

The Gram-negative bacterium *Bordetella pertussis* (*B. pertussis*) causes the severe respiratory infection pertussis, so called whooping cough. Despite a vaccine can prevent disease outbreak, it remains important to understand pathogenicity, as pertussis has resurged in the past years [120,121]. NK cells have been also shown to contribute to the clearance of *B. pertussis*, as disruption of IFN-γ production in the mouse model exacerbated disease progression [122,123]. During infection, *B. pertussis* activates the NLRP3 inflammasome in human macrophages, which results in caspase-mediated IL-18 and IL-1β release [124]. IL-18-mediated NK cell activation promotes the secretion of proinflammatory cytokines such as IFN-γ [125,126] and an enhanced proinflammatory response against the pathogen [124].

### 4.2. ILC2

#### Pneumoniae

By focusing on ILC2 in the lungs, some important functions have been described in a mouse model of *Pneumoniae* infection. IL-13 produced by ILC2 mediates the early polarization of alveolar macrophages into an IL-13-dependent anti-inflammatory M2 phenotype, which plays an important role in the maintenance of lung homeostasis. *IL13^−/−^* mice showed an accelerated immune response and increased bacterial clearance after infection with *S. pneumoniae* [115]. Taken together, ILC2 shape the alveolar macrophage phenotype by promoting a quiescent steady state immune environment, which, however, in turn delays the immune response to *S. pneumoniae.*

### 4.3. ILC3

#### 4.3.1. Pneumoniae

Many studies have also described the importance of IL-22 and IL-17 produced by ILC3 as the key cytokines in bacterial-derived pneumonia [127,128,129]. Particularly, ILC3 rapidly accumulate in the lung, providing the only source of IL-22 during *S. pneumoniae* infection [130]. Upon infection, ILC3 are activated by DCs in a MyD88-dependent manner. Administration of the Toll-like receptor 5 agonist flagellin, which has been shown to activate ILCs and to stimulate cytokine production [131,132], enhanced IL-22 production by ILC3, leading to improved protection from *S. pneumoniae* infection [130]. Gray et al. demonstrated the importance of IL-22-producing ILC3 for resistance to pneumonia in neonates [133]. In their study, intestinal commensal bacteria were crucial for the CD103^+^CD11b^+^ DCs-dependent recruitment of IL-22-producing ILC3 into the lung in order to protect the mice from *S. pneumoniae* infection [133]. Thus, disruption of commensal bacteria impeded the migration of ILC3, leading to increased pathogen susceptibility. Susceptibility could be reversed by administration of exogenous IL-22 or ILC3 transfer. In contrast to the above mentioned studies, clearance of antibiotic-resistant clinical *K. pneumoniae* strains did not depend on IL-22 production [118]. Xiong et al. recently demonstrated the importance of ILC3 as a source of IL-17 in *K. pneumoniae* infection [118]. Here, they described a positive feed-back loop in which TNF-producing inflammatory monocytes were rapidly recruited to the lungs and increased IL-17-producing ILC3. This further enhanced monocyte-mediated bacterial uptake and elimination. Bacterial clearance was reduced in ILC-depleted *Rag2^−/−^* mice. Furthermore, IL-17 and IL-22 are crucial for prevention of chronic airway infection with *P. aeruginosa*, probably by orchestrating neutrophil recruitment [134,135].

Antibiotic resistance is a worldwide challenge for the treatment of bacterial pathogens and has led to the necessity for developing alternative medications. A recent study aimed to unravel the involved host factors responsible for carbapenem-resistant *K. pneumoniae* sequence type 258 (ST258) infection [136]. By using single cell RNA sequencing, the authors identified distinct clusters of IFN-γ-producing NK cells and Il17a^+^Il22^+^ICOS^+^ ILC3 to be critical for the host resistance in this infection. However, these studies did not unequivocally demonstrate the involvement of ILC3.

#### 4.3.2. *Mycobacterium Tuberculosis*

Tuberculosis is an infectious disease caused by *Mycobacterium tuberculosis* and is still the leading cause of death by a single infectious agent [137]. Immune deficient patients in particular show a severe course of disease, illustrating the importance of the innate and adaptive immune systems for disease protection [138].

Recently, a protective role of ILCs in *M. tuberculosis* infection has been demonstrated. During acute infection, the number of ILCs in the peripheral blood of patients was reduced and ILC1 and ILC3, but not ILC2, were restored after clearance of the infection. Despite of ILC depletion in the peripheral blood, studies using mouse models further revealed the accumulation of ILC3 in the lungs of infected mice. This is an essential action for the recruitment of alveolar macrophages and further containment of the infection. Conversely, depletion of ILC3 was associated with impaired immune control of *M. tuberculosis* as a result of reduced production of IL-17 and IL-22 [139].

The generation of functional lymphoid follicles is an essential mechanism in immune defense. The chemokine C-X-C motif chemokine ligand 13 (CXCL13)/C-X-C motif chemokine receptor 5 (CXCR5) interaction induces lymphocyte recruitment and generation of inducible bronchus-associated lymphoid tissues (iBALT) [140]. Both CXCL13 and CXCR5 were upregulated in lungs during *M. tuberculosis* infection [139,140]. This upregulation of CXCR5 on circulating ILCs reveals the importance of the CXCL13/CXCR5-axis for the migration of ILCs and further localization of ILC3 in protective lymphoid follicles within granulomas in the lung [139]. In summary, these studies demonstrate an important protective role of ILC3 in *M. tuberculosis* infection. Additional studies in animal models and data from human are needed to verify these hypotheses and to obtain more detailed insights into the role of ILCs in *M. tuberculosis.*

## 5. Infections of the Skin

### ILC2

In addition to the mucosal surfaces, the skin is one of the largest organs harboring an enormous number of bacteria. ILC2 are the predominant ILC subgroup in the skin, where they contribute to maintenance of skin homeostasis [141]. Human studies of patients suffering from atopic dermatitis (AD) and studies in mice with experimental AD revealed increased numbers of ILC2 and amphiregulin expression in the lesional skin [17,142,143,144]. Atopic dermatitis is accompanied by an increased prevalence of *Staphylococcus aureus* [145]. Recently Hardman et al. showed that human skin ILC2 can express CD1 group 1 protein CD1a upon TSLP activation [146]. CD1a expressing ILC2 were able to sense *S. aureus* via presenting endogenous lipid antigens to CD1a-reactive T cells in a phospholipase 2 (PLA2G4A)-dependent way, eventually promoting skin inflammation [146].

## 6. Systemic Consequences of Bacterial Infections—The Role of ILCs in Sepsis

Sepsis and septic shock can be a severe consequence of a bacterial infection. Sepsis is newly defined as a dysregulated host-response to an infection, which leads to life-threatening organ dysfunction due to a lack of immune homeostasis [147,148]. The initial phase of sepsis is characterized by an augmented activation of the innate immune system leading to the “cytokine storm” and ending up in multiple organ failure [149,150]. ILCs mainly reside in tissues close to the mucosal barrier; however, they are also present in human peripheral blood [149]. Recently, still controversial results have been reported that describe the distribution of circulating ILC subsets in septic patients. Cruz-Zárate et al. reported a significant decrease in ILC1 and ILC3 in peripheral blood of such patients, which was related to the expression of an apoptotic marker on the ILC surface [151]. In contrast, Carvelli et al. did not detect a difference in total ILC cell numbers, but reported an increase in ILC1 and decrease in ILC3 in the peripheral blood of patients with septic shock [149]. They further hypothesized the possible importance of ILC plasticity and an ILC3-ILC1 shift as a reason for the changes in ILC numbers. The differences between these studies might be due to differences in the clinical study conditions, thus, more studies are needed to verify the role of circulating ILCs in sepsis.

Multiple organ failure is one of the main causes of mortality in sepsis. Recently, several groups have begun to examine the contribution of ILCs in secondary organs during sepsis. Chun et al. used a murine model of acute septic shock by cecal ligation and puncture to study the contribution of ILC2 in different organs [152]. The study revealed a rapid increase of ILC2 in the small intestine and peritoneal cavity and a decrease in the liver 24 h after the septic insult. This was accompanied with an increased concentration of IL-13 in the peritoneal fluid. Moreover, mice lacking IL-13-producing cells were shown to have a survival advantage, ameliorated tissue injury and reduced IL-10 concentration in peritoneal fluid [152]. The same sepsis mouse model provided evidence that the number of ILC2 increases in the lung. In fact, lung epithelial cells release increased amounts of IL-33 during sepsis, leading to the expansion of ILC2 via the ST2 receptor [153]. Further experiments revealed that the increased population of ILC2 secrete IL-9, which protects the lung epithelial cells from pyroptosis by attenuating caspase-1 activation [154]. These data suggest a tissue-protective mechanism by ILC2 that negatively regulates lung inflammation following sepsis. These studies demonstrate a complex contribution of ILCs in sepsis with potential organ site-specific effects.

## 7. Discussion

**Figure 1:**
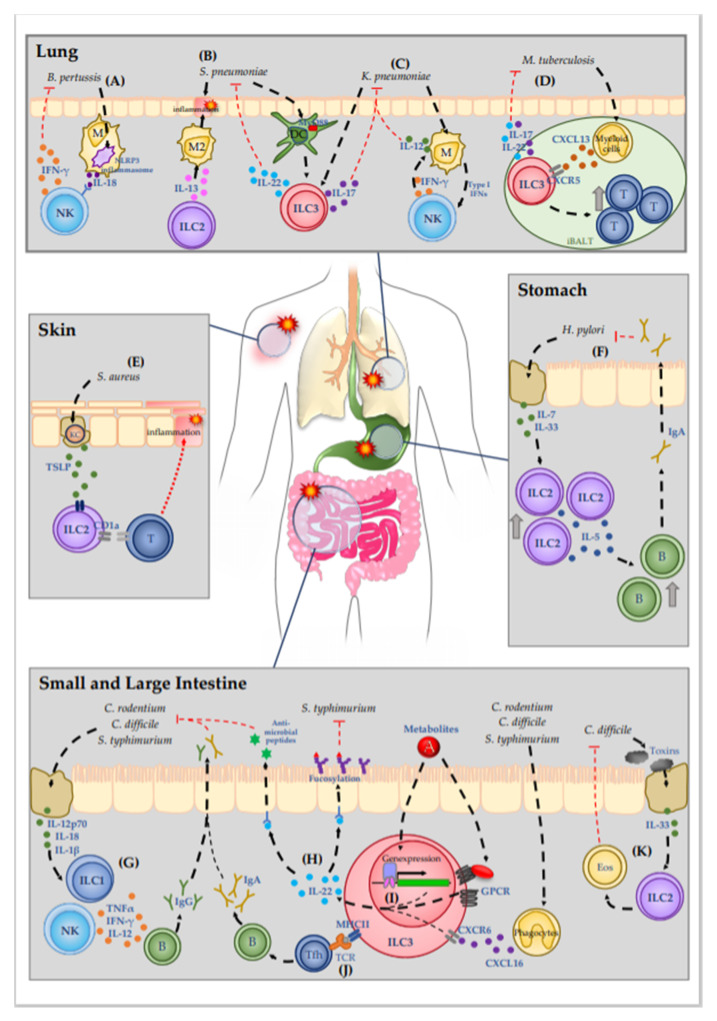
Molecular mechanisms of ILC-mediated immune defense against bacterial infections.Mucosal tissues are sites that are especially prone to bacterial infection due to the high prevalence of pathogenic bacteria. (**A**) *B. pertussis* causes the severe respiratory infection pertussis. Detection of *B. pertussis* by macrophages initiates IL-18 release via NLPR3 inflammasome activation, which promotes IFN-γ release from NK cells. (**B**,**C**) In the lung pneumonia can be caused by the pathogens *S. pneumoniae* and *K. pneumoniae*. (**B**) ILC-2-drived IL-13 mediates early polarization of alveolar macrophages into the M2 phenotype. The quiescent immune environment delays the immune response against *S. pneumoniae*. *S. pneumoniae* is further detected by DC, which then activate ILC3 in a MyD88-dendent manner, resulting in the production of IL-22. (**C**) ILC3 are also activated during *K. pneumoniae* infection, increasing IL-17 expression. A mutual interaction of macrophages and NK cells results in IL-12 release from macrophages that plays a critical role in *K. pneumoniae* containment. (**D**) *M. tuberculosis* infection induces CXCL13 secretion by myeloid cells in the lymphoid tissue. CXCL13 activates ILC3 via CXCR5, inducing the secretion of IL-22 and IL-17. Moreover, activated ILC3 promotes lymphocyte recruitment to the iBALT site, thus inhibiting infection progression. (**E**) Patients with atopic dermatitis are susceptible for *S. aureus* infection. Lesional keratinocytes release TSLP, which increases CD1a expression on ILC2, which activates CD1a-reactive T cells and leads to the recruitment of further immune cells. (**F**) *H. pylori* infection in the stomach leads to epithelial cell damage, thus inducing release of IL-7 and IL-33. Enhanced cytokine secretion boosts ILC2 recruitment and activation and simultaneously enhances the expression of IL-5. IL-5 promotes the recruitment of B cells and concomitant production of IgA. In the stomach lumen, IgA neutralizes the pathogen, thus decreasing bacterial burden in the tissue. (**G**–**K**) In the intestinal tract, pathogens like *C. rodentium*, *C. difficile,* and *S. typhimurium* cause inflammation with various symptoms. (**G**) Damage to epithelial cells induces release of interleukins such as IL-12p70, IL-18 and IL-1β, thus activating ILC1. ILC1-derived TNF-α, IFN-γ, and IL-12 promote the generation of pathogen specific IgG. (**H**–**J**) The most well-studied ILC subgroup in terms of bacterial infections is ILC3. (**H**) Activated ILC3 mediate bacterial infection protection mainly via the secretion of IL-22, which initiates the secretion of antimicrobial peptides from epithelial cells as well as epithelial cell fucosylation, thus impeding bacterial colonization. (**I**) IL-22 expression by ILC3 can be induced by various stimuli: By metabolites, which derive from the gut lumen and activate GPCR receptors or directly modulate gene expression of IL-22 promoting genes, or by the activation of CXCR6 via phagocyte-derived CXCL16. (**J**) Moreover, MHC class II expression on ILC3 allows the direct presentation of pathogen-derived peptide antigens to T cells. promoting the generation of pathogen-specific IgA. (**K**) *C. difficile*-secreted toxins induce epithelial cell damage, resulting in IL-33 release. IL-33 activates ILC2, which promote the recruitment of eosinophils, thereby supporting the clearance of the pathogen and the healing of the epithelial layer. Abbreviations: DC—dendritic cells; M2—type 2 macrophages; T—T cells; KC—Keratinocytes; B—B cells; Eos—Eosinophils; GPCR—G-protein-coupled receptors.

ILCs play an essential role in the maintenance of tissue homeostasis, which is regulated by various microorganisms, especially in tissues at mucosal surfaces (Figure 1). In recent years, many reports revealed the importance of ILCs for host defense mechanisms against bacterial infections, with intensive studies on ILCs in the gastrointestinal tract, as indicated in Table 1. ILC3 and ILC1 are well-known to be involved in the defense against pathogens. However, the latest reports further demonstrated that the ILC2 subset should not be neglected in studies of bacterial infections. Therefore, the currently available information reveals that an intricate network of all ILC subsets is involved in the repulsion of pathogens. Several studies using different bacterial infection models pointed out the specific roles of individual ILC subtypes or the simultaneous involvement of different subtypes. Moreover, cytokine release seems to be specific for the respective bacteria.

The capability of ILCs to change their transcription profiles, and thus undergo a switch within the subsets, makes it even more difficult to understand the complex molecular mechanisms involved in ILC responses. This underlines the importance of further studies to carefully perform ILC subset classification and the necessity for further studies to clarify plasticity promoting factors.

In the last decades, treatment of bacterial infections generally included the use of antibiotics, thereby forcing bacteria to evolve mechanisms to escape the treatment. Thus, antibiotic resistance is currently an increasing concern worldwide. As ILCs are involved in early bacterial defense mechanisms, they might represent a new target for supportive therapy. Similar challenges arise for the treatment of sepsis in the ICU, where a reliable therapy is still lacking. Therefore, the development of new treatment methods is urgently needed. Based on data from recent studies, ILCs might represent a promising target for such new treatment methods. Overall, additional studies are required to confirm and further unravel the detailed regulatory mechanisms of ILCs in bacterial infection and sepsis. Human studies are especially needed to provide a reliable basis for potential treatment development.

## Figures and Tables

**Table 1:** Participation of innate lymphoid cell (ILC) subgroups in bacterial infection defense. Abbreviations: MHCII—major histocompatibility complex class II; VitA—Vitamin A/retinol; RA—retinoic acid; GPCR—G-protein-coupled receptors.

Organ	Bacteria	ILC Subgroup	Cytokines and Involved Signaling Pathways		Effect on Host	References
Intestine	*Citrobacter rodentium*	ILC1	*Activation:*	?		[39,40]
			*Production:*	TNF-α, IFN-γ, IL-12	protective	
		ILC3	*Activation:*	MHCII; GPCR (Ffar2, GPR183); Ahr; VitA/RA; CXCL16/CXCR6		[60,61,72,73,77,78,79,80,82,83,84,85,86,87,88,89,90,91,93,94,95,96,97]
			*Production:*	IL-22	protective	
	*Clostridium difficile*	ILC1	*Activation:*	?		[43]
			*Production:*	IFN-γ	protective	
		ILC2	*Activation:*	IL-33		[57]
			*Production:*	?	protective	
		ILC3	*Activation:*	IL-1β		[43,105]
			*Production:*	IL-22	protective	
	*Salmonella typhimurium*	ILC1	*Activation:*	IL-12p70, IL-18, IL-1β		[36,45,48]
			*Production:*	IFN-γ	protective	
		ILC3	*Inhibition:*	VitA/RA	harmful	[71,88,106,107,108,110]
			*Activation:*	?		
			*Production:*	IL-22	protective	
Stomach	*Helicobacter pylori*	ILC2	*Activation:*	IL-33, IL-7		[56,58]
			*Production:*	IL-5	protective	
Lung	*Klebsiella pneumoniae*	NK	*Activation:*	type I IFNs		[120]
			*Production:*	IFN-γ	protective	
		ILC3	*Activation:*	?		[119,137]
			*Production:*	IL-17	protective	
	*Streptococcus pneumoniae*	ILC2	*Activation:*	?		[116]
			*Production:*	IL-13	harmful	
		ILC3	*Activation:*	?		[131,132,133,134]
			*Production:*	IL-22	protective	
	*Pseudomonas aeruginosa*	ILC3	*Activation:*	?		[135,136]
			*Production:*	IL-17, IL-22	protective	
	*Bordetella pertussis*	NK	*Activation:*	IL-18		[123,124,125]
			*Production:*	IFN-γ	protective	
	*Mycobacterium tuberculosis*	ILC3	*Activation:*	CXCL13/CXCR5		[140,141]
			*Production*	IL-22, IL-17	protective	
Skin	*Staphylococcus aureus*	ILC2	*Activation:*	TSLP; CD1a		[147]
			*Production:*	?	harmful	
